# A Bedside Prediction Score for Strangulation in Adults With Acute Mechanical Intestinal Obstruction: A Prospective Cohort Study in Taiz, Yemen

**DOI:** 10.7759/cureus.112348

**Published:** 2026-07-09

**Authors:** Zaki N Nasr, Shaheed A Alaamry, Sina Al-Nomi, Samer S Al Bothaigi, Omar M Saif

**Affiliations:** 1 Department of General Surgery, Faculty of Medicine and Health Sciences, Taiz University, Taiz, YEM; 2 Department of General Surgery, Al-Jumhuri General Teaching Hospital, Taiz, YEM

**Keywords:** acute intestinal obstruction, acute mechanical bowel obstruction, adhesive intestinal obstruction, bowel strangulation, clinical prediction model, computed tomography, emergency surgery, prediction of bowel strangulation, resource-limited setting, yemen

## Abstract

Background

Early recognition of bowel strangulation in acute mechanical intestinal obstruction (AMIO) is critical but remains difficult when computed tomography (CT) is unavailable, delayed, or not immediately actionable. This study aimed to develop and internally validate a CT-independent bedside prediction score for estimating the risk of bowel strangulation in adults with AMIO in Taiz, Yemen.

Materials and methods

This 12-month prospective observational cohort study, with diagnostic and prediction-model components, enrolled consecutive adults with suspected AMIO at Al-Thawra Hospital and Al-Jumhuri General Teaching Hospital in Taiz, Yemen. Clinical, laboratory, imaging, operative, and outcome data were recorded prospectively. Final strangulation was defined by operative evidence of bowel ischemia, gangrene, necrosis, nonviability, or resection for compromised bowel; successful conservative resolution without later evidence of bowel compromise was classified as non-strangulated. A CT-independent Strangulated Intestinal Obstruction (SIO) score was derived from a five-predictor logistic model and internally validated with 1,000 bootstrap resamples.

Results

Of 230 adults, 212/230 (92.2%) underwent surgery, and 109/212 (51.4%) had intraoperative strangulation. The complete-case prediction cohort included 226 patients. The final score retained pain changing to persistent, abdominal distension, tenderness grade, neutrophilia greater than 75%, and systemic inflammatory response syndrome (SIRS) status. The apparent area under the receiver operating characteristic curve (AUC) was 0.953, and the optimism-corrected area under the curve was 0.947. At a score of 7 or higher, sensitivity was 100/109 (91.7%), specificity was 92/117 (78.6%), positive predictive value was 100/125 (80.0%), negative predictive value was 92/101 (91.1%), and accuracy was 192/226 (85.0%). Observed strangulation risk was 1/76 (1.3%) in the low-risk category, 25/63 (39.7%) in the intermediate-risk category, and 83/87 (95.4%) in the high-risk category.

Conclusion

The score showed promising internal performance as an early triage and escalation aid in this resource-limited pathway. It should support, rather than replace, senior surgical judgment and requires external validation before routine use.

## Introduction

Acute mechanical intestinal obstruction (AMIO) is a familiar emergency general surgical problem. At the bedside, however, the diagnosis of obstruction is often not the most difficult part of the encounter. The decision that matters sooner is whether the obstructed segment is still viable or has begun to strangulate. After vascular compromise begins, the process may pass through venous congestion, mural edema, bowel ischemia, necrosis, perforation, sepsis, and death [1-2]. Symptoms and signs usually establish the obstructive syndrome, but early clinical assessment can look much the same in uncomplicated obstruction, closed-loop obstruction, volvulus, malignant obstruction, and strangulated hernia [3].

Strangulation is better approached as a physiological transition unfolding over time rather than as a single visible event. Rising intraluminal pressure and bowel-wall tension impair venous and lymphatic drainage before arterial inflow is necessarily lost. Clinically, that transition is usually inferred from fragments of evidence: colicky pain becomes persistent, distension becomes harder to ignore, tenderness develops or worsens, neutrophilia appears, and systemic physiology begins to drift. A bedside score that combines these signals is therefore more consistent with the biology of the condition than reliance on one symptom, one sign, or one laboratory value.

Current surgical guidance supports nonoperative management only in selected patients, particularly in adhesive small-bowel obstruction, when peritonitis, strangulation, and bowel ischemia are absent or not suspected [4-5]. That qualification is essential. Observation is safe only when repeated examination, resuscitation, timely imaging where needed, and rapid operative escalation can be delivered. These conditions are less secure when patients present late, operating-room access is limited, or computed tomography (CT) is selective, delayed, unaffordable, non-contrast, or not reported in time for the operative decision.

The case-mix of AMIO is not uniform across health systems. Adhesions dominate many high-income small-bowel obstruction series, whereas hernia-related obstruction, volvulus, malignancy, and delayed presentation still contribute substantially in many low- and middle-income settings [6-8]. Yemeni studies describe a similarly mixed etiologic pattern and a heavy operative burden [9-12]. In Taiz, emergency surgical care is delivered within a conflict-affected, resource-constrained environment, making early bedside risk stratification a practical clinical need rather than a purely academic exercise [13-14].

Preoperative recognition of strangulation remains unreliable. Sarr et al. showed that conventional combinations of fever, tachycardia, leukocytosis, continuous pain, and peritoneal signs could not safely exclude strangulation [15]. Later prediction studies improved risk assessment by combining clinical, laboratory, and radiological variables [16-21]. Their usefulness at the first surgical decision point may still be limited when a tool depends on CT, advanced biomarkers, or selected adhesive small-bowel obstruction cohorts, especially if CT has not yet been obtained or the report does not comment on bowel viability in a way that can guide action.

CT remains valuable for defining anatomy and danger signs, including the obstruction level, transition point, closed-loop configuration, volvulus, reduced enhancement, mesenteric congestion, pneumatosis, portal venous gas, and perforation [22-26]. However, in a delayed or selective imaging pathway, the immediate surgical question is how to communicate and act upon strangulation risk before ideal information is available. The primary objective was to derive and internally validate a CT-independent bedside score for estimating final strangulation risk during early emergency surgical assessment. Secondary objectives were to describe the diagnostic and operative profile of AMIO in this pathway and to evaluate the diagnostic contribution and limitations of CT when imaging was obtained.

## Materials and methods

Study design and setting

This prospective observational cohort study combined diagnostic-pathway and prediction-model components and was conducted from July 1, 2024, through June 30, 2025, at Al-Thawra Hospital and Al-Jumhuri General Teaching Hospital in Taiz, Yemen. Both are public referral hospitals providing emergency general surgical care to Taiz City and surrounding districts. Routine care was not altered. Investigation choices, operative timing, and treatment pathways remained the responsibility of the attending surgical teams.

The design deliberately followed the emergency pathway as it was delivered rather than imposing an ideal protocol. A diagnostic test has clinical value only if it is available, documented, interpretable, and acted on before the decision it is meant to influence. A CT scan performed after an operation has effectively been decided, or a report that confirms obstruction without addressing bowel viability, cannot be treated as equivalent to an immediately available structured CT assessment. Diagnostic-pathway variables were therefore kept separate from prediction-model variables.

Participants

Consecutive adults aged 18 years or older were eligible if they presented with suspected AMIO. Clinical suspicion required an acute obstructive syndrome, usually abdominal pain with vomiting, distension, constipation, obstipation, or failure to pass flatus, supported by radiology, operative findings, or successful conservative resolution without an alternative diagnosis, consistent with established clinical descriptions and management guidance [1-5]. Exclusion criteria were paralytic ileus, pseudo-obstruction, non-mechanical obstruction, age younger than 18 years, or unavailable core outcome data. Three analysis populations were specified: the full enrolled cohort for pathway description, the complete-case cohort for prediction modeling, and the CT-imaged subgroup for CT diagnostic-accuracy analysis.

Analysis of populations and missing data

The full cohort comprised all registered eligible adults and was used to describe patient flow, operative burden, and diagnostic-pathway features. Records with missing neutrophil percentage were excluded from the complete-case prediction cohort because neutrophilia was a prespecified candidate predictor and imputation was not planned for this derivation analysis. The CT subgroup included only patients who actually underwent CT and had the relevant feature assessable. Unperformed or non-evaluable imaging was left as unavailable rather than coded as a negative result.

Data sources and predictor definitions

Prospective data were entered on a structured case-report form and cross-checked against emergency notes, admission records, vital-sign charts, laboratory reports, radiology reports, operative notes, anesthesia records, discharge summaries, and available follow-up documentation. The final predictor domains were retained because they were available early, biologically plausible for bowel compromise, and independent of CT: pain changing from colicky or intermittent to persistent, abdominal distension, tenderness grade, neutrophilia greater than 75%, and systemic inflammatory response syndrome (SIRS) status. Tenderness grade was coded as no tenderness, tenderness without rebound, or rebound tenderness. SIRS was coded using conventional temperature, heart-rate, respiratory-rate, and white-cell criteria [27]. Routine hematologic markers have also contributed to previous strangulation prediction models [16,19,21].

Predictor selection combined prespecified clinical reasoning, early bedside availability, biological plausibility for bowel compromise, and multivariable assessment. Candidate predictors were considered only if they could be documented during early emergency surgical assessment before CT was performed or reported. CT variables were excluded from the prediction model by design. The retained predictors were selected because they were clinically interpretable, available in the intended pathway, and independently associated with final strangulation status in the multivariable model.

Outcome definition

Final strangulation status was the primary outcome. In operated patients, the operative note provided the reference standard. Strangulation was defined as intraoperative bowel ischemia, gangrene, necrosis, nonviability, or bowel resection for compromised vascularity, consistent with established descriptions of strangulated obstruction [1,2,15]. Patients managed without surgery were classified as non-strangulated only when in-hospital clinical resolution occurred without later evidence of bowel compromise. This pragmatic outcome classification matches the real emergency pathway, but it also introduces partial verification bias, which is considered in the limitations.

CT assessment

CT findings were analyzed only in CT-imaged patients with evaluable documentation. Patients who did not undergo CT were not treated as CT-negative. Recorded CT variables included bowel dilatation, transition-point information where documented, small-bowel fecalization, mesenteric edema, engorged mesenteric veins, whirl sign, pneumatosis intestinalis, closed-loop obstruction or volvulus, bowel-wall, mesenteric, or portal venous gas, and explicit radiological diagnosis of strangulation [22-26]. CT was treated as an anatomical investigation and, when timely and actionable, a clinical override tool; it was not built into the bedside score.

CT was performed according to routine emergency radiology practice rather than a research-mandated imaging protocol. Intravenous contrast was used when clinically feasible and not contraindicated; oral contrast was used selectively according to surgeon-radiologist judgment and patient condition. CT reports were generated during routine care by the available radiology service, and the study did not include blinded central re-reading or formal interobserver agreement testing.

CT variables were abstracted from the radiology report and, when available, from documented surgeon-radiologist communication. Imaging signs were defined clinically as follows: transition-point information referred to a documented site or level of caliber change; closed-loop obstruction or volvulus referred to an explicit report of a closed-loop configuration or rotational obstruction/volvulus; the whirl sign was recorded separately when explicitly described; pneumatosis intestinalis and bowel-wall, mesenteric, or portal venous gas were recorded when explicitly described; and radiologic strangulation was recorded only when the report explicitly suggested ischemia, nonviability, strangulation, or equivalent wording [22-26]. Because CT availability, contrast use, and reporting detail varied across the pathway, CT was analyzed as a separate diagnostic layer and was not incorporated into the bedside score.

Clinical risk categories

Predicted probabilities were grouped into three clinical zones: low risk (less than 0.10), intermediate risk (0.10-0.29), and high risk (0.30 or greater). The low zone was intended to support monitored observation only in clinically stable patients with no override criterion. The intermediate zone was intended to prompt senior review and reassessment. The high zone was intended to accelerate escalation and operative preparedness. These zones are clinical descriptors from a derivation analysis, not externally validated management rules.

Model development and score translation

Multivariable binary logistic regression estimated the probability of final strangulation in the complete-case cohort. The model was deliberately kept CT-independent so that it could be used before CT was performed or reported. The final logistic-regression linear predictor was written in plain text as follows: log odds of strangulation = -4.549 + 2.372 × persistent pain + 1.442 × abdominal distension + 1.365 × tenderness grade + 2.203 × neutrophilia + 1.021 × SIRS. Persistent pain, abdominal distension, neutrophilia, and SIRS were coded as 1 when present and 0 when absent; tenderness grade was coded as 0 for no tenderness, 1 for tenderness without rebound, and 2 for rebound tenderness. Regression coefficients were converted into integer bedside weights for the simplified Strangulated Intestinal Obstruction (SIO) score: persistent pain, 4 points; abdominal distension, 3 points; tenderness grade, 0, 2, or 5 points; neutrophilia, 4 points; and SIRS, 2 points. The total score ranged from 0 to 18. A sensitivity analysis replaced the ordinal tenderness term with two categorical indicators, using no tenderness as the reference category, and also examined a binary tenderness specification. The score was designed to support triage and escalation, not to function as an automatic operative rule.

Calibration was examined because a model may rank patients accurately yet misestimate absolute risk. Brier score, calibration intercept, calibration slope, and grouped observed-versus-predicted risk were assessed alongside the area under the receiver operating characteristic curve (AUC). For AMIO triage, discrimination is useful only if predicted risks also sit at clinically sensible escalation thresholds.

Statistical analysis and internal validation

All p-values were two-sided. Diagnostic performance was interpreted descriptively because the study was designed to derive and internally validate a bedside tool rather than compare treatment strategies. Confidence intervals were reported for threshold-level model performance, and exact score-level risks were interpreted cautiously when cell counts were small. Data management and primary checks were performed using IBM SPSS Statistics for Windows, Version 27.0 (Released 2019; IBM Corp., Armonk, NY, USA). The final reproducible analyses were conducted in jamovi, version 2.7 (The jamovi Project, Sydney, Australia), using the R 4.5 computational environment (R Foundation for Statistical Computing, Vienna, Austria).

The model probability threshold of 0.30 was selected on clinical grounds to prioritize sensitivity and a low false-negative rate because a missed strangulation carries greater clinical risk than a false-positive escalation. Apparent performance was summarized with AUC, Brier score, calibration measures, sensitivity, specificity, predictive values, accuracy, and likelihood ratios. Bootstrap internal validation used 1,000 resamples, refitting the five-predictor model and testing it against the original complete-case dataset. Reporting was informed by TRIPOD+AI, STARD 2015, and STROCSS 2024 [28-30].

Ethical approval and informed consent

The study protocol was approved by the Ethics Committee for Scientific Research, Faculty of Medicine and Health Sciences, Taiz University, Taiz, Yemen (reference ECTU2024/20-6; approval date January 4, 2024). Administrative permission was obtained from Al-Thawra Hospital and Al-Jumhuri General Teaching Hospital, Taiz, Yemen. Individual informed consent was not obtained. The study was observational, did not alter routine clinical care, and used de-identified clinical data. No identifying patient information appears in the manuscript, tables, figures, or supplementary material.

Data availability

The de-identified analytic dataset, data dictionary, and analysis code are retained by the investigators and may be made available to qualified researchers on reasonable request, subject to Taiz University approval and applicable patient-confidentiality requirements.

## Results

Cohort flow

**Figure 1:**
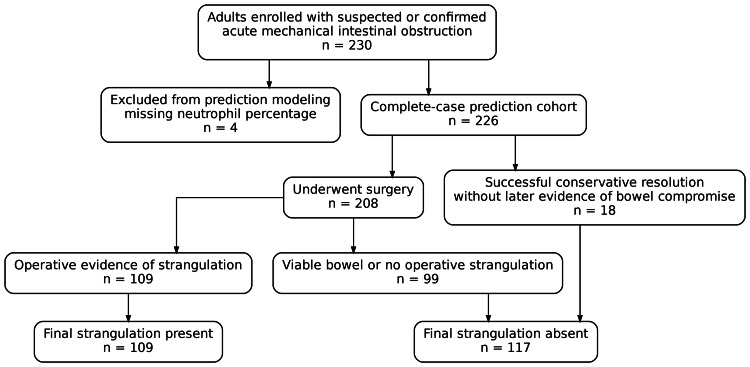
Participant Flow Into the Complete-Case Prediction Cohort Four patients were excluded because the neutrophil percentage was missing. Final strangulation was defined by operative evidence among operated patients. Successful conservative resolution without later evidence of bowel compromise supported classification as non-strangulated.

The study enrolled 230 adults with suspected or confirmed AMIO. Of these, 212/230 (92.2%) underwent surgery, and intraoperative strangulation was identified in 109/212 operated patients (51.4%). Four records were excluded from prediction modeling because neutrophil percentage was missing, leaving 226 complete-case observations. In that complete-case cohort, 208 patients underwent surgery and 18 achieved conservative resolution. Final strangulation was present in 109/226 complete-case patients (48.2%) (Figure 1).

Baseline and presenting features

Median age was 35 years (interquartile range, 25-54), and 144/226 patients (63.7%) were male. Previous abdominal surgery was recorded in 106/226 patients (46.9%). Age, sex, and previous abdominal surgery showed no material difference by final strangulation status. At first assessment, final strangulation was associated with pain changing to persistent, abdominal distension, increasing tenderness grade, leukocytosis, neutrophilia greater than 75%, and SIRS status (Table 1).

**Table 1:** Baseline and First-Assessment Characteristics by Final Strangulation Status Values are n (%) unless otherwise stated. The p-value for tenderness grade is the global comparison across its three levels; category rows therefore have no separate p-values. A single hyphen (-) indicates not applicable. *Statistically significant p-value (p < 0.05). IQR: interquartile range; SIRS: systemic inflammatory response syndrome.

Variable	Overall (n = 226)	No final strangulation (n = 117)	Final strangulation (n = 109)	p-value
Age, years, median (IQR)	35 (25-54)	35 (25-50)	36 (27-57)	0.154
Male sex	144 (63.7)	73 (62.4)	71 (65.1)	0.772
Previous abdominal surgery	106 (46.9)	56 (47.9)	50 (45.9)	0.868
Pain changed to persistent	87 (38.5)	9 (7.7)	78 (71.6)	<0.001*
Abdominal distension	164 (72.6)	75 (64.1)	89 (81.7)	0.005*
Tenderness grade, global comparison	-	-	-	<0.001*
No tenderness	80 (35.4)	69 (59.0)	11 (10.1)	-
Tenderness without rebound	90 (39.8)	41 (35.0)	49 (45.0)	-
Rebound tenderness	56 (24.8)	7 (6.0)	49 (45.0)	-
Leukocytosis	102 (45.1)	20 (17.1)	82 (75.2)	<0.001*
Neutrophilia >75%	100 (44.2)	12 (10.3)	88 (80.7)	<0.001*
SIRS criteria met	97 (42.9)	26 (22.2)	71 (65.1)	<0.001*

The complete-case cohort served as the principal modeling dataset, while the full cohort was retained for pathway description. Denominators follow the relevant analysis: model performance estimates refer to 226 patients, operative etiology to the 212 patients who underwent surgery, and CT performance to CT-imaged patients only.

Operative management and etiology

Among operated patients, adhesions were the leading etiology, accounting for 89 of 212 cases (42.0%). External or abdominal wall hernia accounted for 56 of 212 cases (26.4%), volvulus for 32 of 212 (15.1%), tumor or malignant obstruction for 26 of 212 (12.3%), and less common causes for the remaining cases. Among patients with strangulation, adhesive obstruction accounted for 49 of 109 (45.0%), volvulus for 22 of 109 (20.2%), hernia-related obstruction for 24 of 109 (22.0%), and tumor or malignant obstruction for 10 of 109 (9.2%).

CT pathway and diagnostic performance

CT was obtained in 131/230 patients (57.0%). Among 129 CT-imaged patients with available contrast documentation, intravenous contrast was used in 101/129 (78.3%) and oral contrast in 42/129 (32.6%). An explicit radiologic diagnosis of strangulation appeared in six CT-imaged patients. Mesenteric edema, engorged mesenteric veins, whirl sign, pneumatosis, closed-loop obstruction or volvulus, bowel-wall, mesenteric, or portal venous gas, and explicit CT diagnosis of strangulation produced no false-positive observations among 75 non-strangulated CT-imaged patients (specificity 75/75, 100.0%). Sensitivities for these signs ranged from 6/56 (10.7%) to 15/56 (26.8%) (Table 2).

**Table 2:** Diagnostic Performance of Computed Tomography Findings Among Imaged Patients Findings not performed or not assessable were not coded as negative. CT: computed tomography; PPV: positive predictive value; NPV: negative predictive value

CT finding	n	Sensitivity (%)	Specificity (%)	PPV (%)	NPV (%)	Accuracy (%)
Small-bowel dilatation >=4 cm	131	57.1	69.3	58.2	68.4	64.1
Dilated loops with thickened valvulae conniventes	131	41.1	97.3	92.0	68.9	73.3
Small-bowel fecalization	131	53.6	90.7	81.1	72.3	74.8
Mesenteric edema	131	26.8	100.0	100.0	64.7	68.7
Engorged mesenteric veins	131	25.0	100.0	100.0	64.1	67.9
Whirl sign	131	10.7	100.0	100.0	60.0	61.8
Pneumatosis intestinalis	131	21.4	100.0	100.0	63.0	66.4
Closed-loop obstruction or volvulus	131	14.3	100.0	100.0	61.0	63.4
Bowel-wall, mesenteric, or portal venous gas	131	10.7	100.0	100.0	60.0	61.8
Explicit CT diagnosis of strangulation	131	10.7	100.0	100.0	60.0	61.8

Final model and bedside score

The final model retained five predictors: persistent pain, abdominal distension, tenderness grade, neutrophilia greater than 75%, and SIRS status (Table 3). Each remained independently associated with final strangulation. Adjusted odds ratios were 10.72 (95% CI, 3.86-29.78) for persistent pain, 9.05 (95% CI, 3.42-23.95) for neutrophilia, 4.23 (95% CI, 1.40-12.81) for abdominal distension, 3.92 per level (95% CI, 2.05-7.46) for tenderness grade, and 2.78 (95% CI, 1.03-7.46) for SIRS.

**Table 3:** Final Multivariable Model and Bedside Score Weights Tenderness grade was entered once as an ordinal predictor coded as 0 (no tenderness), 1 (tenderness without rebound), and 2 (rebound tenderness). The three tenderness score rows show bedside score translation only and were not fitted as separate regression terms. A single hyphen (-) indicates not applicable. *Statistically significant p-value (p < 0.05). CI: confidence interval; OR: odds ratio; SIO: strangulated intestinal obstruction; SIRS: systemic inflammatory response syndrome

Predictor or score component	Coefficient	Adjusted OR	95% CI lower	95% CI upper	p-value	SIO points
Pain changed to persistent: yes vs no	2.372	10.72	3.86	29.78	<0.001*	4
Abdominal distension: yes vs no	1.442	4.23	1.40	12.81	0.011*	3
Tenderness grade: per one-level increase	1.365	3.92	2.05	7.46	<0.001*	See score rows
Tenderness score: no tenderness	-	-	-	-	-	0
Tenderness score: tenderness without rebound	-	-	-	-	-	2
Tenderness score: rebound tenderness	-	-	-	-	-	5
Neutrophilia >75%: yes vs no	2.203	9.05	3.42	23.95	<0.001*	4
SIRS criteria met: yes vs no	1.021	2.78	1.03	7.46	0.043*	2
Intercept	-4.549	0.01	0.00	0.05	<0.001*	-

For the bedside SIO score, persistent pain received 4 points, abdominal distension 3 points, tenderness grade 0, 2, or 5 points, neutrophilia greater than 75% 4 points, and SIRS 2 points. Observed risk stayed low at scores of 0-4 and was very high at scores of 11-18, although exact-score estimates were unstable where cell counts were small. The selected threshold of 7 or higher closely matched the high-sensitivity 0.30 predicted-probability threshold.

A sensitivity analysis examined the ordinal assumption for tenderness. Using no tenderness as the reference category, tenderness without rebound was associated with final strangulation (adjusted OR 2.90, 95% CI 1.01-8.37, p = 0.048), while rebound tenderness showed a stronger association (adjusted OR 16.32, 95% CI 4.40-60.61, p < 0.001). The categorical specification did not materially improve fit compared with the primary ordinal model (likelihood-ratio p = 0.489; AUC 0.954 vs 0.953; Brier score 0.086 for both). A binary present/absent specification performed less well. The ordinal term was therefore retained in the primary model (Table 4).

**Table 4:** Sensitivity Analysis of Tenderness Specification and Category-Specific Effects The categorical and binary models were sensitivity analyses. The p-value of 0.489 is from the likelihood-ratio comparison of the categorical and ordinal models. Category-specific adjusted odds ratios are from the categorical sensitivity model. The ordinal model was retained for parsimony and preservation of clinical severity information. A single hyphen (-) indicates not applicable. *Statistically significant p-value (p < 0.05). AIC: Akaike information criterion; AUC: area under the receiver operating characteristic curve; aOR: adjusted odds ratio; CI: confidence interval

Analysis/comparison	Estimate or parameters	Model df	AIC	AUC	Brier score	p-value
Ordinal grade (primary model)	1 parameter	5	137.39	0.953	0.086	-
Categorical grade	2 indicators	6	138.92	0.954	0.086	0.489
Binary present/absent	1 parameter	5	145.35	0.946	0.092	-
Tenderness without rebound vs no tenderness	aOR 2.90 (95% CI 1.01-8.37)	-	-	-	-	0.048*
Rebound tenderness vs no tenderness	aOR 16.32 (95% CI 4.40-60.61)	-	-	-	-	<0.001*

Model performance and risk categories

**Figure 2:**
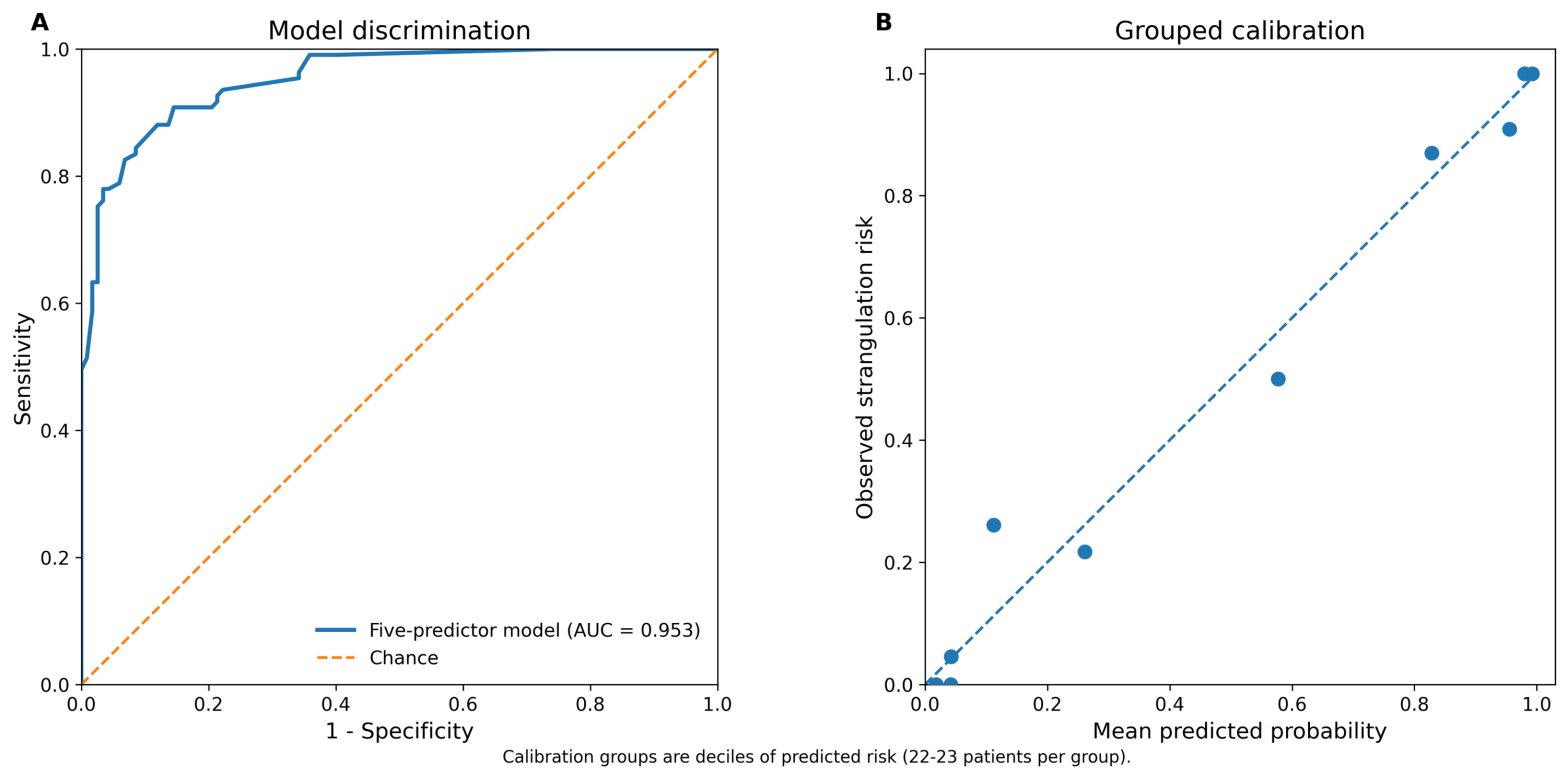
Model Discrimination and Grouped Calibration Panel A shows the receiver operating characteristic curve for the point-based Strangulated Intestinal Obstruction (SIO) score. The apparent area under the curve (AUC) of the underlying logistic model is shown for reference. Panel B shows observed versus mean predicted strangulation risk by the prespecified probability zone. Both panels are based on the derivation cohort; discrimination and calibration require external reassessment.

The apparent AUC was 0.953, with a Brier score of 0.086. At the clinically selected 0.30 probability threshold, approximated by an SIO score of 7 or higher, sensitivity was 100/109 (91.7%; 95% CI, 85.0-95.6), specificity was 92/117 (78.6%; 95% CI, 70.4-85.1), positive predictive value was 100/125 (80.0%; 95% CI, 72.1-86.1), negative predictive value was 92/101 (91.1%; 95% CI, 83.9-95.2), and accuracy was 192/226 (85.0%; 95% CI, 79.7-89.0). Bootstrap correction yielded an AUC of 0.947, a Brier score of 0.095, and a calibration slope of 0.916. Observed strangulation risk was 1/76 (1.3%) in the low SIO category, 25/63 (39.7%) in the intermediate category, and 83/87 (95.4%) in the high category (Tables 5-6 and Figure 2).

**Table 5:** SIO Score Categories and Observed Strangulation Risk The categories are derivation-cohort descriptors and are not externally validated treatment rules. Clinical danger signs override the numerical score. SIO: strangulated intestinal obstruction

SIO category	n	Events/n	Observed risk	Proposed interpretation
0-4 points, low	76	1/76	1.30%	Observation may be reasonable only if clinically stable and no override criterion is present.
5-10 points, intermediate	63	25/63	39.70%	Senior surgical review, repeat examination, resuscitation, and CT when actionable.
11-18 points, high	87	83/87	95.40%	Urgent escalation and preparation for possible operation.

**Table 6:** Threshold Performance and Bootstrap Internal Validation A single hyphen (-) indicates that a confidence interval or optimism-corrected estimate was not available for that measure. Optimism-corrected values were obtained from 1,000 bootstrap resamples. Internal validation estimates optimism but does not establish external validity. AUC: area under the receiver operating characteristic curve; CI: confidence interval

Performance measure	Apparent estimate	95% CI lower	95% CI upper	Optimism-corrected estimate
AUC	0.953	-	-	0.947
Brier score	0.086	-	-	0.095
Calibration slope	1.000	-	-	0.916
Sensitivity at threshold 0.30	91.70%	85.00%	95.60%	91.00%
Specificity at threshold 0.30	78.60%	70.40%	85.10%	78.10%
Positive predictive value	80.00%	72.10%	86.10%	79.40%
Negative predictive value	91.10%	83.90%	95.20%	90.30%
False-negative rate	8.30%	4.40%	15.00%	9.00%
Accuracy	85.00%	79.70%	89.00%	-

## Discussion

Principal findings

The most striking feature of this cohort was the severity of AMIO reaching referral surgical care in Taiz. Surgery was performed in 212 of 230 patients (92.2%), and 109 of the 212 operated patients (51.4%) had intraoperative strangulation. These proportions should not be interpreted as community prevalence. They may reflect referral delay, admission selection, a mixed operative etiology, and substantial surgeon concern at presentation. For the clinicians receiving these patients, the practical question was whether the available bedside evidence justified faster escalation before bowel compromise became irreversible.

The etiologic profile also argues against treating AMIO in this setting as a narrow adhesive small-bowel obstruction problem. Adhesions were the commonest cause, but hernia-related obstruction, volvulus, and malignancy together made up a large part of the operative workload. That pattern fits broader evidence showing that obstruction etiology changes across regions and health systems [6-8], and it is consistent with Yemeni reports describing mixed causes rather than a purely adhesive small-bowel obstruction profile [9-12]. Clinically, the difference matters. A tight band may produce closed-loop physiology; a tender irreducible hernia can strangulate before laboratory markers become dramatic; volvulus threatens both lumen and mesentery; malignant obstruction brings a separate operative and oncological burden.

CT was most persuasive when it showed danger. In the CT-imaged subgroup, pneumatosis, closed-loop configuration, whirl sign, mesenteric vascular changes, and explicit radiological suspicion of strangulation were highly specific and should carry weight in senior review and operative preparedness. Their absence, however, was not sufficiently reassuring here. This agrees with radiological literature showing that CT improves anatomical assessment while individual ischemic signs vary in sensitivity [22-26]. It also reflects a pathway reality: CT can change management only if the patient reaches the scanner, contrast is feasible, bowel viability is addressed in the report, and the result is available before the operative decision has effectively been made.

Interpretation of predictors and threshold

The retained predictors are recognizable bedside surgical findings rather than abstract statistical constructs. Pain changing from colicky or intermittent to persistent suggests movement away from simple peristaltic obstruction toward sustained mural tension, mesenteric traction, venous congestion, or early peritoneal irritation. Distension reflects mechanical load, luminal accumulation, third spacing, and duration of obstruction. Tenderness grade preserves the clinical gradient of abdominal examination rather than flattening it into a binary label. In the primary model, tenderness was fitted once as an ordinal 0-2 variable; the categorical sensitivity analysis did not improve model fit, and the 0/2/5 score weights are a bedside translation rather than separate fitted effects. Neutrophilia and SIRS add inflammatory and physiological context. No single feature is dependable enough on its own; the useful signal lies in the pattern formed when they converge.

The chosen threshold followed a safety-oriented rationale. The clinically selected 0.30 probability threshold, approximated by an SIO score of 7 or higher, favored sensitivity because a missed strangulation is generally the more serious error. A false-positive score should trigger senior review, resuscitation, repeat examination, useful imaging when it can be obtained without dangerous delay, and operating-room preparedness; it should not, by itself, mandate laparotomy. A false-negative classification may delay recognition of necrosis, perforation, sepsis, or death. A low score should therefore never be used as a discharge label or as a reason to override senior surgical concern. It has value only when the patient is stable, improving, observed, and free from clinical or radiologic override criteria.

The model's performance should still be read with caution. A bootstrap-corrected AUC of 0.947 indicates strong discrimination in this dataset, and the calibration slope of 0.916 suggests optimism but not severe instability. Internal validation remains internal. A hospital with lower strangulation prevalence, different operative thresholds, faster CT access, or another etiologic mix may see different calibration and predictive values. Before any protocol-level use, external validation should examine AUC, calibration, risk-category behavior, and the clinical details of false-negative cases.

Previous prediction tools provide useful comparisons, but they do not always answer the same question. Some predict the need for operation; others focus on adhesive small-bowel obstruction, irreversible ischemia, or CT-confirmed cases [16-21]. A patient may need a laparotomy after failed conservative management without having non-viable bowel. Conversely, early strangulation may still be reversible if the constricting mechanism is released promptly. The SIO score was developed to support early triage of final strangulation risk in a broad AMIO cohort, rather than to predict all patients who would ultimately require laparotomy or bowel resection. Accordingly, it should be regarded as a pre-CT bedside risk-stratification aid that complements, but does not replace, timely and actionable CT when such imaging is immediately available and safe to obtain.

In practice, the SIO score should therefore be interpreted as a pre-CT bedside triage aid and should complement, not replace, timely and actionable CT when CT is immediately available and safe to obtain. A low score may support monitored observation only when the patient is stable and has no peritonitis, shock, painful irreducible hernia, deterioration, or high-risk CT features. An intermediate score should raise concern, prompt senior review, and lead to repeat examination after resuscitation. A high score should shorten avoidable delay and accelerate operating-room preparation, particularly when waiting for CT would be unsafe. Peritonitis, shock, painful irreducible hernia, clinical deterioration, perforation, reduced enhancement, closed-loop obstruction, pneumatosis, portal venous gas, or overriding surgeon concern should supersede the numerical score.

Operationally, the score may be most useful as a common language at admission. A resident can document pain evolution, distension, tenderness grade, neutrophil percentage, and SIRS status; the resulting score gives the consultant, anesthesiologist, and operating-room team a compact description of risk. That role is deliberately modest. Better documentation and clearer prioritization are useful, but they do not replace serial abdominal examination, resuscitation, radiology discussion, or the operative responsibility of the surgeon at the bedside.

Strengths and limitations

Several strengths support the relevance of the findings to emergency surgical practice. Data were collected prospectively during routine care, and operative assessment of bowel viability served as the reference standard in operated patients. CT was analyzed as a separate diagnostic layer rather than being treated as negative when it had not been performed. The prediction model used bedside variables and routine hematology, which makes it feasible in constrained pathways. Separating diagnostic accuracy, prediction modeling, and triage interpretation also reduces the risk of presenting a score as though it were a treatment rule.

A prediction model and a pathway rule are not the same thing. The model estimates risk from early variables. The pathway determines what that risk should trigger in the presence of resuscitation needs, anesthetic readiness, CT availability, senior review, and operating-room access. A high score should make delay harder to justify; it should not remove operative judgment. A low score should support observation only when observation is genuinely safe and reassessment is dependable.

The limitations are substantial. This was a derivation study conducted in two public referral hospitals in one city, where the case-mix may have influenced model performance. Both hospitals receive advanced and high-concern AMIO cases, and the cohort had a high operative rate and a high strangulation event rate. This spectrum may have increased apparent discrimination and may not reflect lower-acuity settings, earlier presentations, pathways with broader nonoperative management, or systems with faster access to standardized CT.

Non-operated patients did not have operative verification; those who resolved with conservative treatment were therefore classified pragmatically as non-strangulated, introducing partial verification bias. CT use was selective, and several CT signs depended on routine documentation rather than standardized acquisition, blinded central radiology review, or interobserver assessment. Although the sample size supported a five-predictor model, etiology-specific performance could not be estimated reliably. Timing data were insufficient for detailed time-to-event modeling.

The score has not been externally validated and should not be adopted as a protocol-level mandate without prospective validation and, if necessary, recalibration. External validation should test both discrimination and calibration in cohorts with different AMIO prevalence, operative thresholds, etiologic mix, imaging availability, and clinical pathways. The 0.30 escalation threshold was clinically selected and was not supported by public preregistration, so threshold-based performance also requires prospective confirmation.

Any threshold also has to be interpreted within the resources around it. In a system with immediate CT, continuous monitoring, and dependable operating-room access, escalation may mean rapid imaging followed by a planned operation. In a constrained pathway, the same risk estimate may need to trigger direct consultant review and avoidance of non-essential diagnostic delay. The SIO score should therefore be viewed internationally as a tool for decision-making under uncertainty, not as a Yemen-specific shortcut or a substitute for established surgical judgment.

Future research

Subsequent research should externally validate the SIO score in contemporary Taiz cohorts and in other Yemeni or regional emergency surgical pathways. Validation should include calibration plots, decision-curve analysis, detailed review of false-negative cases, etiology-specific performance, and process outcomes such as time to senior review, time to CT, time to operative decision, bowel resection, morbidity, mortality, and length of stay. A further priority is to evaluate bedside scoring together with structured CT reporting and direct communication between surgeons and radiologists.

## Conclusions

In this prospective emergency surgical cohort from Taiz, Yemen, strangulation was frequent among operated adults with AMIO. A CT-independent model combining pain progression, abdominal distension, tenderness grade, neutrophilia, and SIRS status showed strong apparent discrimination and favorable optimism-corrected internal performance. The simplified SIO score separated patients into clinically distinct risk groups and may support early triage, senior review, resuscitation, and operative preparedness.

The score is not an operative rule. Peritonitis, shock, a painful irreducible hernia, clinical deterioration, and unequivocal ischemic CT findings should override the numerical estimate. Because the model was derived in two public referral hospitals with a high event rate and selective CT use, external validation and, if needed, recalibration are required before routine protocol-level implementation.

## References

[1] Catena F, De Simone B, Coccolini F, Di Saverio S, Sartelli M, Ansaloni L (2019). Bowel obstruction: a narrative review for all physicians. World J Emerg Surg.

[2] Cappell MS, Batke M (2008). Mechanical obstruction of the small bowel and colon. Med Clin North Am.

[3] Long B, Robertson J, Koyfman A (2019). Emergency medicine evaluation and management of small bowel obstruction: evidence-based recommendations. J Emerg Med.

[4] Ten Broek RP, Krielen P, Di Saverio S (2018). Bologna guidelines for diagnosis and management of adhesive small bowel obstruction (ASBO): 2017 update of the evidence-based guidelines from the World Society of Emergency Surgery ASBO working group. World J Emerg Surg.

[5] Maung AA, Johnson DC, Piper GL (2012). Evaluation and management of small-bowel obstruction: an Eastern Association for the Surgery of Trauma practice management guideline. J Trauma Acute Care Surg.

[6] Markogiannakis H, Messaris E, Dardamanis D (2007). Acute mechanical bowel obstruction: clinical presentation, etiology, management and outcome. World J Gastroenterol.

[7] Soressa U, Mamo A, Hiko D, Fentahun N (2016). Prevalence, causes and management outcome of intestinal obstruction in Adama Hospital, Ethiopia. BMC Surg.

[8] Falola AF, Dada OS, Ndong A, Akande DG (2024). Etiology and management outcomes of adult mechanical bowel obstruction in Nigeria: a systematic review and meta-analysis. World J Surg.

[9] Al-Bahlooli SH, Abdurabo OY, Al-Bahlooly AHM, Shaban MAM, Naeem AY, Al-Shujaa MA (2018). Pattern of intestinal obstruction at Hajjah Governorate in Yemen. Ain Shams J Surg.

[10] Ghabisha S, Ahmed F, Altam A, Hassan F, Badheeb M (2023). Small bowel obstruction in virgin abdomen: predictors of surgical intervention need in a resource-limited setting. J Multidiscip Healthc.

[11] Matar AI, Obadiel YA, Jowah HM (2025). Acute mechanical bowel obstruction: a comprehensive analysis of clinical presentation, etiology, surgical management, and postoperative outcomes in a Yemeni cohort. J Emerg Med Trauma Acute Care.

[12] Alashaby SS, Gilan WM, Al-Absy TA, Al-Magedi AA (2026). Etiology, clinical profile, management, and outcomes of intestinal obstruction in a resource-limited setting: a prospective study. Sci Rep.

[13] (2026). Hospitals and staff under constant attack in Taiz, Yemen. https://www.msf.org/hospitals-and-staff-under-constant-attack-taiz-yemen.

[14] (2026). Yemen: WHO Health Emergency Appeal 2024. https://www.who.int/publications/m/item/yemen-who-2024-health-emergency-appeal.

[15] Sarr MG, Bulkley GB, Zuidema GD (1983). Preoperative recognition of intestinal strangulation obstruction. Prospective evaluation of diagnostic capability. Am J Surg.

[16] Jancelewicz T, Vu LT, Shawo AE, Yeh B, Gasper WJ, Harris HW (2009). Predicting strangulated small bowel obstruction: an old problem revisited. J Gastrointest Surg.

[17] Schwenter F, Poletti PA, Platon A, Perneger T, Morel P, Gervaz P (2010). Clinicoradiological score for predicting the risk of strangulated small bowel obstruction. Br J Surg.

[18] Huang X, Fang G, Lin J, Xu K, Shi H, Zhuang L (2018). A prediction model for recognizing strangulated small bowel obstruction. Gastroenterol Res Pract.

[19] Bouassida M, Laamiri G, Zribi S (2020). Predicting intestinal ischaemia in patients with adhesive small bowel obstruction: a simple score. World J Surg.

[20] Zielinski MD, Eiken PW, Bannon MP, Heller SF, Lohse CM, Huebner M, Sarr MG (2010). Small bowel obstruction-who needs an operation? A multivariate prediction model. World J Surg.

[21] Murao S, Fujino S, Danno K (2023). Ischemia prediction score (IsPS) in patients with strangulated small bowel obstruction: a retrospective cohort study. BMC Gastroenterol.

[22] Mallo RD, Salem L, Lalani T, Flum DR (2005). Computed tomography diagnosis of ischemia and complete obstruction in small bowel obstruction: a systematic review. J Gastrointest Surg.

[23] Millet I, Taourel P, Ruyer A, Molinari N (2015). Value of CT findings to predict surgical ischemia in small bowel obstruction: a systematic review and meta-analysis. Eur Radiol.

[24] Nakashima K, Ishimaru H, Fujimoto T (2015). Diagnostic performance of CT findings for bowel ischemia and necrosis in closed-loop small-bowel obstruction. Abdom Imaging.

[25] Paulson EK, Thompson WM (2015). Review of small-bowel obstruction: the diagnosis and when to worry. Radiology.

[26] Nielsen LB, Ærenlund MP, Alouda M (2023). Real-world accuracy of computed tomography in patients admitted with small bowel obstruction: a multicentre prospective cohort study. Langenbecks Arch Surg.

[27] Bone RC, Balk RA, Cerra FB (1992). Definitions for sepsis and organ failure and guidelines for the use of innovative therapies in sepsis. The ACCP/SCCM Consensus Conference Committee. Chest.

[28] Collins GS, Moons KG, Dhiman P (2024). TRIPOD+AI statement: updated guidance for reporting clinical prediction models that use regression or machine learning methods. BMJ.

[29] Bossuyt PM, Reitsma JB, Bruns DE (2015). STARD 2015: an updated list of essential items for reporting diagnostic accuracy studies. BMJ.

[30] Rashid R, Sohrabi C, Kerwan A, Franchi T, Mathew G, Nicola M, Agha RA (2024). The STROCSS 2024 guideline: strengthening the reporting of cohort, cross-sectional, and case-control studies in surgery. Int J Surg.

